# Flux variability scanning based on enforced objective flux for identifying gene amplification targets

**DOI:** 10.1186/1752-0509-6-106

**Published:** 2012-08-21

**Authors:** Jong Myoung Park, Hye Min Park, Won Jun Kim, Hyun Uk Kim, Tae Yong Kim, Sang Yup Lee

**Affiliations:** 1 Metabolic and Biomolecular Engineering National Research Laboratory, Department of Chemical and Biomolecular Engineering (BK21 program), Center for Systems and Synthetic Biotechnology, Institute for the BioCentury, Korea Advanced Institute of Science and Technology (KAIST), Daejeon, 305-701, Republic of Korea; 2 BioInformatics Research Center, KAIST, Daejeon, 305-701, Republic of Korea; 3 Department of Bio and Brain Engineering and BioProcess Engineering Research Center, KAIST, Daejeon, 305-701, Republic of Korea

**Keywords:** Flux variability scanning based on enforced objective flux, Grouping reaction constraints, Putrescine, *Escherichia coli*

## Abstract

**Background:**

In order to reduce time and efforts to develop microbial strains with better capability of producing desired bioproducts, genome-scale metabolic simulations have proven useful in identifying gene knockout and amplification targets. Constraints-based flux analysis has successfully been employed for such simulation, but is limited in its ability to properly describe the complex nature of biological systems. Gene knockout simulations are relatively straightforward to implement, simply by constraining the flux values of the target reaction to zero, but the identification of reliable gene amplification targets is rather difficult. Here, we report a new algorithm which incorporates physiological data into a model to improve the model’s prediction capabilities and to capitalize on the relationships between genes and metabolic fluxes.

**Results:**

We developed an algorithm, flux variability scanning based on enforced objective flux (FVSEOF) with grouping reaction (GR) constraints, in an effort to identify gene amplification targets by considering reactions that co-carry flux values based on physiological omics data *via* “GR constraints”. This method scans changes in the variabilities of metabolic fluxes in response to an artificially enforced objective flux of product formation. The gene amplification targets predicted using this method were validated by comparing the predicted effects with the previous experimental results obtained for the production of shikimic acid and putrescine in *Escherichia coli*. Moreover, new gene amplification targets for further enhancing putrescine production were validated through experiments involving the overexpression of each identified targeted gene under condition-controlled batch cultivation.

**Conclusions:**

FVSEOF with GR constraints allows identification of gene amplification targets for metabolic engineering of microbial strains in order to enhance the production of desired bioproducts. The algorithm was validated through the experiments on the enhanced production of putrescine in *E. coli*, in addition to the comparison with the previously reported experimental data. The FVSEOF strategy with GR constraints will be generally useful for developing industrially important microbial strains having enhanced capabilities of producing chemicals of interest.

## Background

One of the most ambitious goals in metabolic engineering is the design of biological systems based on *in silico* predictions using mathematical models. The advent of high-throughput technologies and the completion of genome sequencing for many organisms have led to an explosion of systems-wide biological data [1,2]. Genome-scale stoichiometric models of the increasing number of microorganisms and mammalian cells have been developed at the moment [3,4]. Some of such models have been used to identify gene knockout targets for the efficient production of important industrial chemicals, including amino acids [5,6] and chemicals that are conventionally derived from petroleum [7-9]; other such models have been used to identify drug targets in pathogens [10-12]. In modeling and simulation approaches, target reactions whose knockout is predicted to overproduce the chemical of interest can be easily tested experimentally by deleting the corresponding genes in the microbial host.

Increasing the expression levels of the relevant genes has also been successfully employed for the overproduction of target chemicals [13,14]. To avoid unnecessarily massive experiments to be performed, several computational algorithms have been devised in an effort to reveal the relationship between metabolic reactions and the biological properties of interest [15-27]; however, the identification of gene amplification targets is more complicated than the identification of gene knockout targets; hence, correlations among the genes, mRNAs, transcriptional or translational regulations, proteins, and metabolic fluxes must be carefully examined. Genome-scale metabolic models that rely on constraints-based flux analysis without additional physiological information are limited in their ability to describe the complex nature of biological systems, particularly biological phenomena beyond metabolism. Several systematic methods have been developed to overcome such limitations: flux variability analysis (FVA) [17,19-21], flux coupling analysis [16-18], flux sensitivity analysis [15], flux response analysis [26], OptReg [22], genetic design through local search [25], OptForce [27], and flux scanning based on enforced objective flux (FSEOF) [23]. In particular, FSEOF is a method that first scans and searches for variations in the metabolic fluxes in response to the enforced fluxes directed towards a target product. Reactions were then selected as amplification targets, the flux values of which increased in accordance with the enforced fluxes toward the production of a target chemical. This method was experimentally validated by identifying amplification targets that improved the production of lycopene in *Escherichia coli*[23]. These approaches demonstrated that incorporating physiological constraints during the model simulation are critical to identifying trustworthy gene amplification targets, but much improvement is still needed [24,28]. One of the major problems is the existence of a too large flux solution space in optimization problems.

**Figure 1:**
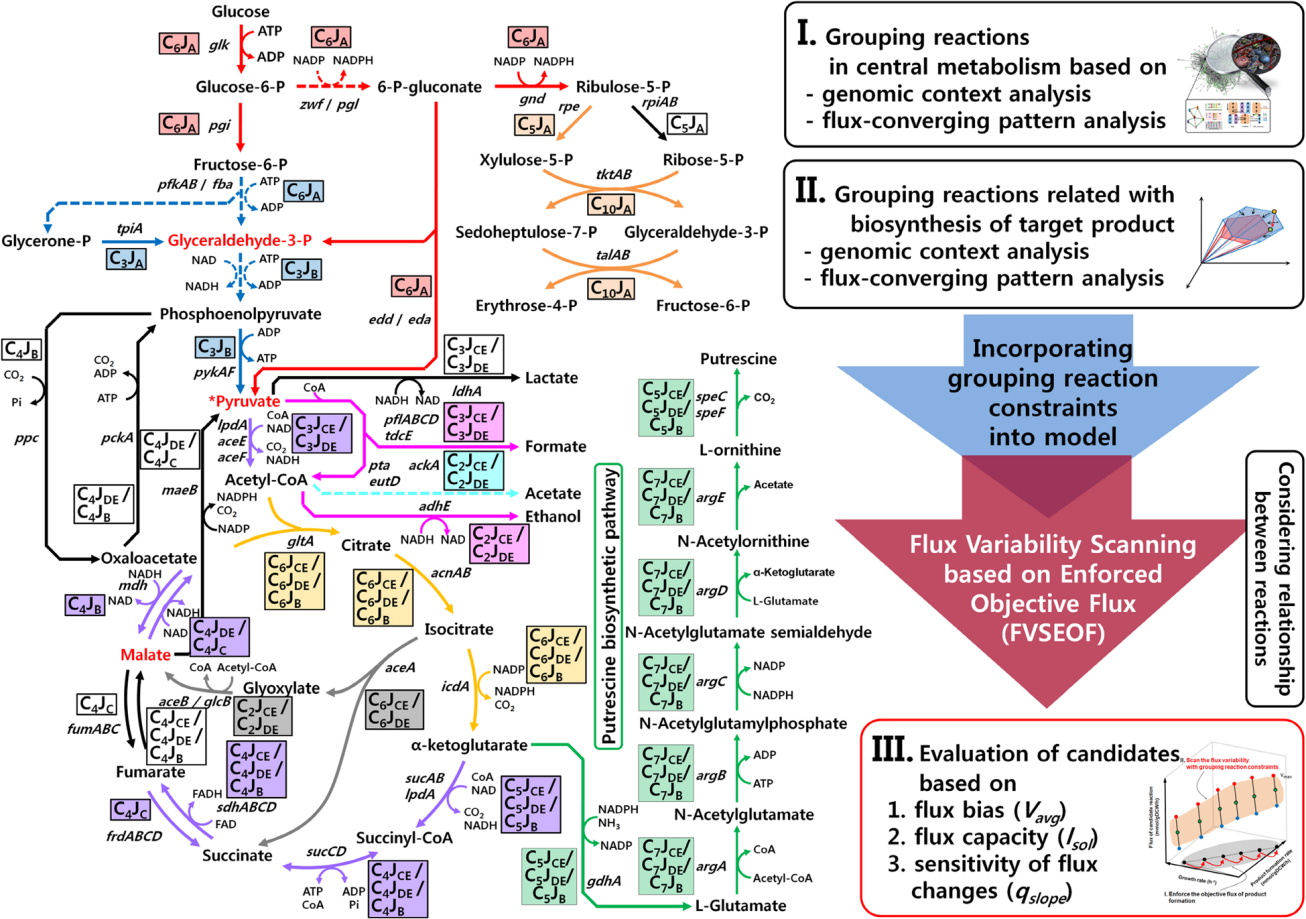
**Schematic illustration of the FVSEOF method with GR constraints.** Functionally grouped reactions were considered based on genomic context and flux-converging pattern analyses obtained from the STRING database. FVSEOF was then performed under GR constraints to identify gene amplification candidates for the production of a target chemical. The candidates were evaluated based on the model predictions and additional criteria of the flux bias ( *V*_*avg*_) and the slope of the flux changes (*q*_*slope*_). Each rectangle containing a *C*_*x*_*J*_*y*_ index and a line with different colors defines the reaction groups that are likely on or off simultaneously, as determined by genomic context and flux-converging pattern analyses. The *C*_*x*_*J*_*y*_ index for each reaction is determined by flux-converging pattern analysis. *C*_*x*_ and *J*_*y*_ denote the total number of carbon atoms in metabolites that participate in each reaction and the type of fluxes through the flux-converging metabolites from a carbon source, respectively. The red metabolites indicate flux-converging metabolites. The flux-converging metabolites indicate metabolites at which two pathways split by another metabolite recombine. For example, glyceraldehyde-3-phosphate converges the fluxes split by the fructose-bisphosphate aldolase from the fructose-6-phosphate. The flux-converging metabolites categorize *J*_*y*_ into four types, indicated as *J*_*A*_, *J*_*B*_, *J*_*C*_, and *J*_*D*_. Each subscript of *J*_*y*_ denotes the number of flux-converging metabolites that are passed zero, one, two, or three times, respectively, for a given flux from a carbon source. The subscript *E* is specially denoted to indicate the fluxes derived from pyruvate. The values of *C*_*x*_*J*_*y*_ for each reaction were assigned based on possible flux routes reaching from glucose, and are partitioned by a slash.

In this study, in order to systematically handle the large flux solution spaces, as also revealed in the implementation of FSEOF [23], we considered functionally grouped reactions that simultaneously carry fluxes based on unique features of microbial genomes. Considering such functionally grouped reactions helps reducing the number of and selecting multiple solutions existing for each optimal objective value, enabling to identify more reliable gene amplification targets when combined with FSEOF. Grouped reactions were previously revealed by genomic context and flux-converging pattern analyses as promising constraints [28]. Genomic context analysis interrogates conserved neighborhood, gene fusion, and co-occurrence using a STRING database with the goal of suggesting groups of reaction fluxes that are most likely correlated in their on/off activities [28,29]. Flux-converging pattern analysis further limits the range of possible flux values in a metabolic reaction by examining the number of carbon atoms in metabolites that participate in the reactions and the converging patterns of fluxes from a carbon source (see Methods and Figure 1) [28]. Consequently, flux balance analysis (FBA) with constraints controlling simultaneous on/off activity (*C*_*on/off*_) and the flux scale (*C*_*scale*_) of the metabolic reactions accurately predicted flux distributions in gene knockout mutant strains [28].

Based on these analyses, the grouping reaction (GR) constraints that constrain reactions to co-carry fluxes altogether regardless of the condition were incorporated into the *E. coli* genome-scale metabolic model. The model then facilitated the scanning of changes in the variability among metabolic fluxes using FVA in response to the enforced enhancement of the fluxes toward a target chemical. This newly developed method, called flux variability scanning based on enforced objective flux (FVSEOF) with GR constraints, was employed in this study to identify gene amplification targets for the production of target chemicals. FVSEOF with GR constraints was first validated based on amplification targets reported for the production of shikimic acid and putrescine in *E. coli*, and then further validated by actually engineering *E. coli* for the enhanced production of putrescine based on new amplification targets.

## Methods

### *E. coli* genome-scale metabolic model

EcoMBEL979 was used throughout this study [30], which is a slightly modified version of the genome-scale *E. coli* metabolic network model, *i*JR904 [31]. EcoMBEL979 contains 814 metabolites (144 extracellular metabolites and 670 intermediates) and 979 metabolic reactions, along with a biomass equation derived from the *E. coli* biomass composition [32].

### Constraints-based flux analysis

The stoichiometric relationships among the metabolites and the reactions of the *E. coli* genome-scale metabolic model were balanced under the pseudo-steady state assumption. The balanced reaction model was almost always underdetermined in calculations of the flux distribution due to insufficient measurements of the extracellular fluxes. Thus, the unknown fluxes within the metabolic reaction network were calculated by linear programming-based optimization using an objective function that maximized the growth rate, subject to constraints pertaining to mass conservation and reaction thermodynamics [33], This optimization problem can be mathematically formulated as follows:

(1) $$\sum_{j\in J}S_{\mathrm{ij}}v_{j}=b_{i}$$

(2) $$a_{j}\leq v_{j}\leq \beta_{j}$$

where *S*_*ij*_ represents the stoichiometric coefficient for metabolite *i* in reaction *jν*_*j*_ is the flux of reaction *jJ* is the set of all reactions, and *b*_*i*_ is the net transport flux of metabolite *i*. If this metabolite is an intermediate, *b*_*i*_ is equal to zero. *α*_*j*_ and *β*_*j*_ are the lower and upper bounds of the flux of reaction *j*, respectively. Herein, the flux of any irreversible reaction is considered to be positive; a negative flux indicates the reverse direction of a reaction.

### Grouping reaction (GR) constraints based on the genomic context and flux-converging pattern analyses

The algorithm introduced in this study, FVSEOF with GR constraints, starts with formulation of GR constraints, which are based on the genomic context and flux-converging pattern analyses (Figure 1). Briefly, genomic context and flux-converging pattern analyses aim at grouping functionally related reactions. Such functionally related reactions were constrained to be on or off simultaneously (Figure 1) [28]. First, reactions were grouped using STRING database that performs genomic context analysis, including conserved neighborhood, gene fusion, and co-occurrence [28,29]. Simultaneous on/off constraint (*C*_*on/off*_) can be described as follows:

(3) $$y\left(v_{1}\right)=y\left(v_{2}\right)$$

(4) $$y\left(v_{1}\right)\cdot \alpha_{1}\leq v_{1}\leq y\left(v_{1}\right)\cdot \beta_{1}$$

(5) $$y\left(v_{2}\right)\cdot \alpha_{2}\leq v_{2}\leq y\left(v_{2}\right)\cdot \beta_{2}$$

where *y*( *v*_*1*_) and *y*( *v*_*2*_) indicate binary variables (on or off) of a certain reaction 1 and 2, respectively.

Each reaction is then given a *C*_*x*_*J*_*y*_ index, determined by flux-converging pattern analysis. *C*_*x*_ and *J*_*y*_ denote the total number of carbon atoms in metabolites that participate in each reaction and the number of passing flux-converging metabolites, respectively. Here, it should be noted that cofactors were not considered because the flux scales are controlled by the carbon number of primary metabolites, not cofactors, according to ^13^C-based flux analysis [28]. For *J*_*y*_, the flux-converging metabolites indicate metabolites at which two pathways split by another metabolite converge. *J*_*y*_ has four types, including *J*_*A*_*J*_*B*_*J*_*C*_, and *J*_*D*_, depending on the characteristics of flux-converging metabolites. Subscript of *J*_*y*_ denotes the passing number of flux-converging metabolites, counting zero, one, two, or three times for the flux coming from a carbon source. In some cases, the subscript E is placed next to the subscripts of A, B, C, or D to indicate the fluxes derived from pyruvate, which causes more complex changes in flux distributions. The values of *C*_*x*_*J*_*y*_ for each reaction were assigned based on possible flux routes reaching from glucose, and are partitioned by a slash. Based on this analysis, another constraint *C*_*scale*_, indicating the flux scale of a reaction, can be given to the metabolic reactions. First, terms used to describe the flux scale of the reaction are as follows:

(6) $$C_{\mathrm{xy}}J_{\mathrm{yj}}$$

(7) $$x_{j}=\frac{N_{C,R_{j}}}{2}$$

where $C_{\mathrm{xj}}$ indicates the carbon number involved in a reaction *j*$J_{\mathrm{yj}}$ the number of the passing of the flux through the flux-converging metabolite near reaction *j*, and *N*_*C,Rj*_ the total number of carbon of primary metabolites without cofactors in reaction *j*.

If reaction 1 and 2 were predicted to be in the same functional unit according to the genomic context analysis, and their $C_{x1}J_{y1}$ and $C_{x2}J_{y2}$ are equivalent, *C*_*scale*_ is applied to these two reactions, which is defined as follows:

(8) $$\sqrt{\frac{{\left(\left|v_{1}^{n}\right|-\frac{\left|v_{1}^{n}\right|+\left|v_{2}^{n}\right|}{2}\right)}^{2}+{\left(\left|v_{2}^{n}\right|-\frac{\left|v_{1}^{n}\right|+\left|v_{2}^{n}\right|}{2}\right)}^{2}}{2}}\leq \delta$$

where *v*^*n*^_*1*_ and *v*^*n*^_*2*_ are the normalized flux of reaction 1 and 2, obtained by dividing each reaction flux by the carbon source uptake rate, such as glucose. δ is the constant defining the flux level of reactions in this functional unit; the value of δ is recommended as 0.3.

### Flux variability scanning based on enforced objective flux (FVSEOF) with grouping reaction (GR) constraints

**Figure 2:**
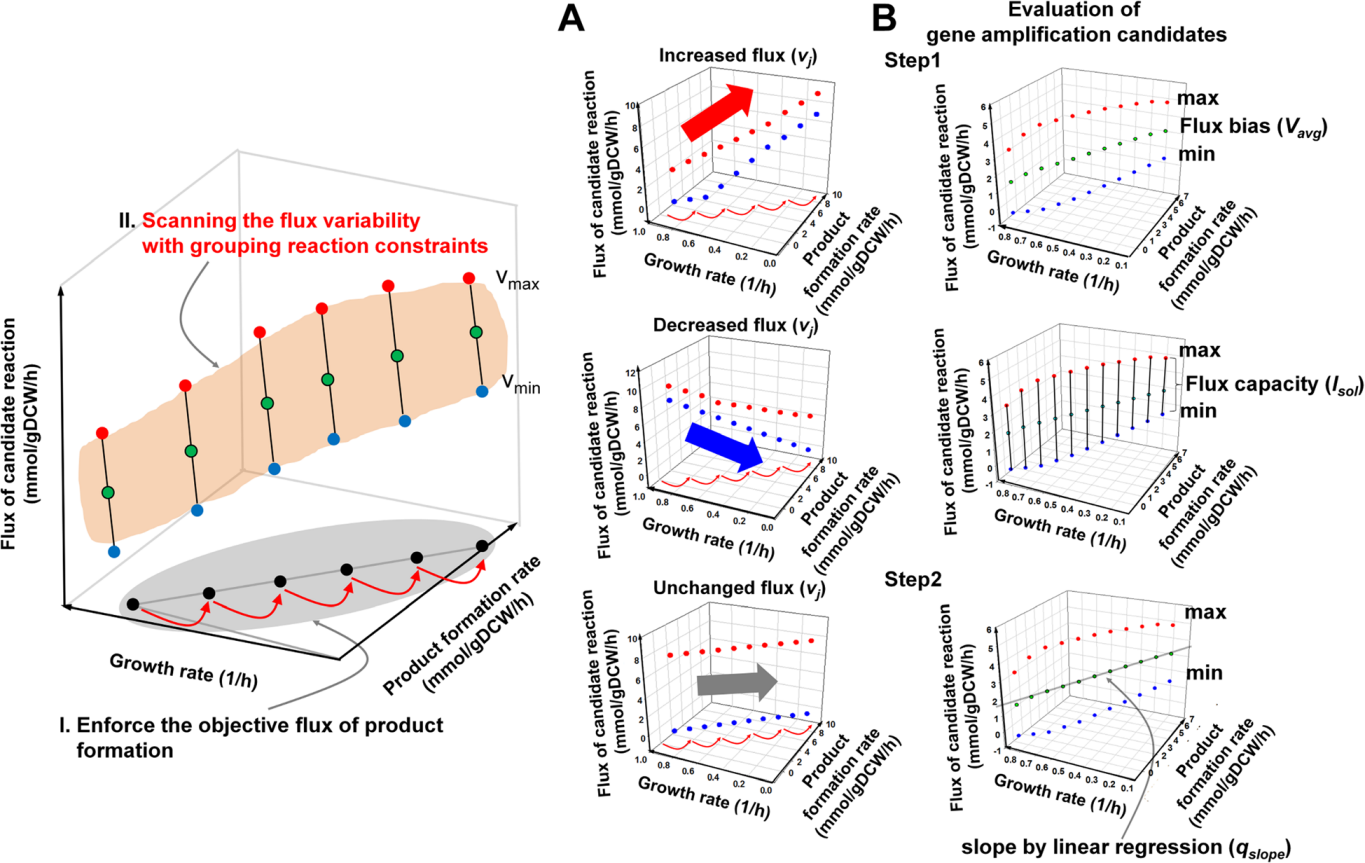
**Framework of the FVSEOF with GR constraints for identifying gene amplification targets that enhance the production of a target product.** The FVSEOF method scans the changes in the metabolic flux variabilities in response to an enhanced flux toward a target product. The method then selects amplification target reactions, the fluxes of which increase in response to the forced increase in the flux toward the target bioproduct. ( **A**) During the FVSEOF implementation, three types of intracellular flux profiles are typically identified: increased, decreased, or unchanged, but oscillatory flux profiles can also be found in some cases. ( **B**) To evaluate the gene amplification candidates, the slope ( *q*_*slope*_) was calculated based on a linear regression between the enforced production rate of a target product and the *V*_*avg*_ values of the candidate reactions. The positive correlation in the slope indicates that the corresponding reaction may be a gene amplification candidate. On the basis of the *q*_*slope*_ values, we considered the sensitivities of the identified gene amplification candidates to the enforced production of a target chemical. A large value of *q*_*slope*_ indicates that the corresponding reaction may be more sensitive to the enforced production of a target chemical, than reactions with smaller *q*_*slope*_ values.

Once grouping reaction constraints are defined, FVSEOF with GR constraints is subsequently performed as follows (Figure 2). First, the initial or theoretical minimum ($v_{target\;product}^{\mathrm{initial}}$) and theoretical maximum ($v_{target\;product}^{\max}$) of the target product formation rates were calculated; these were implemented by setting the objective function as minimizing and maximizing the target product formation rate using constraints-based flux analysis with GR constraints. This can be formulated as follows:

Min/Max $Z\left(v_{target\;product}\right)$ = $v_{target\;product}^{\mathrm{initial}}$ or $v_{target\;product}^{\max}$

(9) $$\text{Subject to}\;\sum_{j\in J}S_{\mathrm{ij}}v_{j}=b_{i}$$

(10) $$l_{i}\leq \sum_{j\in J}S_{\mathrm{ij}}v_{j}\leq u_{i}$$

(11) $$\alpha_{j}\leq v_{j}\leq \beta_{j}$$

(12) $$\beta_{j}=1000\text{mmol}\cdot {\text{g DCW}}^{-1}\cdot {\text{h}}^{-1}$$

(13) $$\alpha_{j}=-1000\text{mmol}\cdot {\text{g DCW}}^{-1}\cdot {\text{h}}^{-1}$$

(14) v mathvariant="italic">carbon mathvariant="italic">uptake=10mmol·g DCW−1·h−1

where $v_{target\;product}^{\mathrm{initial}}$ indicates the initial or minimal point of the flux value constrained for the target bioproduct, while $v_{target\;product}^{\max}$ indicates the maximal flux value for the bioproduct. *l*_*i*_ and *u*_*i*_ are the lower and upper bound for the net transport flux of metabolite *i*, respectively, and $v_{\mathrm{carbon}}^{\mathrm{uptake}}$ is the carbon source uptake rate.

Second, the cell growth rate, *Z*( *v*_*biomass*_), was maximized while gradually increasing the target product formation rate from its initial (or minimal) flux value to its near theoretical maximum: $v_{target\;product}^{\mathrm{enforced}}=v_{target\;product}^{\mathrm{initial}}+\frac{k}{n}\left(v_{target\;product}^{\max}-v_{target\;product}^{\mathrm{initial}}\right)$$K=\left\{k|k=1,2,\cdots ,n-1\right\}$ (n≥10) [23]. The $v_{target\;product}^{\mathrm{enforced}}$ is an additional constraint provided during this stage of the constraints-based flux analysis; it starts with the initial value $v_{target\;product}^{\mathrm{initial}}$ plus one *n*^th^ of the difference between the $v_{target\;product}^{\max}$ and $v_{target\;product}^{\mathrm{initial}}$, and is increased to a value adjacent to $v_{target\;product}^{\max}$ in *k* steps.

Third, FVA was carried out with GR constraints by maximizing or minimizing the fluxes of all intracellular reactions, *Z*( *v*_*intracellular reaction*_), with additional constraints: the enforced production rate of the target bioproduct, which varied from its initial to maximum values in 10 steps, and 95% optimal cell growth rate, *v*_*biomass*_ = 0.95 · *Z*( *v*_*biomass*_)^*opt*^, for each step. The attainable flux ranges of intracellular reactions for each step were subsequently subjected to the targeting criteria introduced in the following section.

FVSEOF with GR constraints was calculated using mixed integer nonlinear programming with the DICOPT solver, subject to the constraints including GR constraints, mass conservation and reaction thermodynamics.

### Flux bias, its slope and flux capacity as targeting criteria

**Figure 3:**
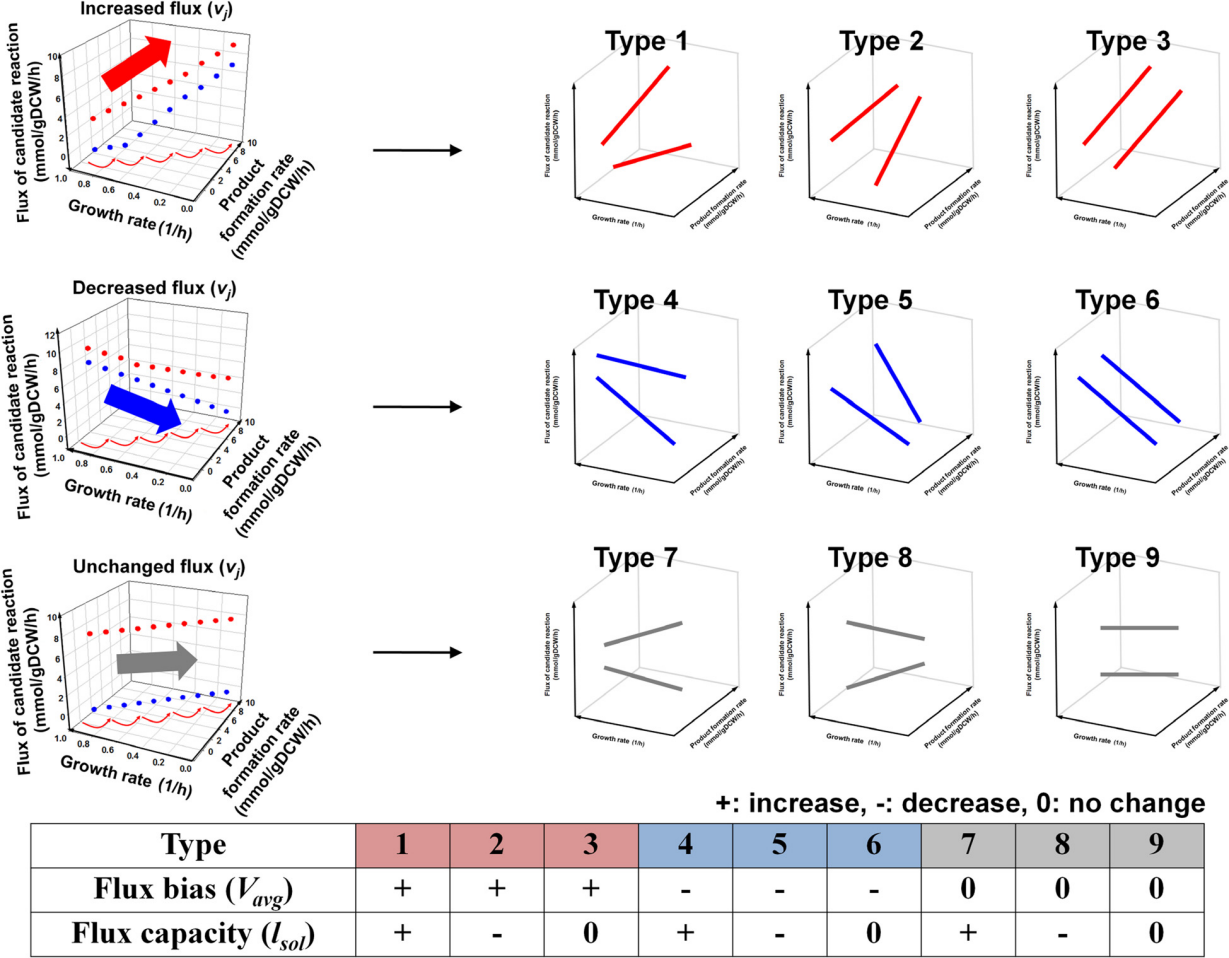
**Nine types of changes in the flux patterns based on combinations of positive and negative changes in*V***_***avg***_**and*l***_***sol***_**.** Types 1, 2, and 3 have the amplification candidates positively correlated with the production of a target chemical. Types 4, 5, and 6 were negatively correlated with the target product. Types 7, 8, and 9 did not show unique patterns with the enforced fluxes towards the target chemical. Nine possible combinations of flux bias ( *V*_*avg*_) and flux capacity (*l*_*sol*_) for each reaction were investigated, and displayed on the bottom of the figure.

Flux bias (*V*_*avg*_), its slope (*q*_*slope*_) and flux capacity (*l*_*sol*_) were employed as targeting criteria for the initial set of gene amplification targets predicted from FVSEOF with GR constraints (Figure 2 and 3). Among them, *V*_*avg*_ and *l*_*sol*_ were determined as follows in order to effectively investigate the changes of flux variabilities for genetic perturbations [28]:

(15) $$V_{\mathrm{avg}}=\frac{V_{\max}^{'}+V_{\min}^{'}}{2}$$

(16) $$l_{\mathrm{sol}}=V_{\max}^{'}-V_{\min}^{'}$$

The ${V^{\prime }}_{\max}$and ${V^{\prime }}_{\min}$indicate the maximal and minimal flux values for a reaction under the given condition. The *l*_*sol*_ indicates the difference between the maximal and minimal flux values for a reaction. *q*_*slope*_ was calculated using linear regression of the flux values for a reaction towards the gradually maximized product formation rate.

### Bacterial strains and plasmids

The *E. coli* strains used in this study are listed in the Additional file 1. The XQ52 strain, a putrescine producer, was used as a base strain [34]. *E. coli* TOP10 was used for gene cloning studies. The plasmid p15SpeC containing a strong *tac* promoter was used as an expression vector. The plasmid p15SpeC was constructed from the pTac15K plasmid by cloning the *speC* gene (encoding ornithine decarboxylase in the putrescine biosynthetic pathway) into the site between the *EcoRI* and *SacI* restriction enzyme sites of pTac15K. The plasmid contained a kanamycin resistance selective marker. Cells were grown in Luria–Bertani (LB) broth or on plates containing appropriate antibiotics at 37°C for the construction of strains and plasmids. Antibiotics were added at following concentrations: 50 μg/mL ampicillin, 25 μg/mL kanamycin, and 35 μg/mL chloramphenicol.

The plasmids used in this study are listed in the Additional file 1. Polymerase chain reaction (PCR) primers for the gene cloning studies conducted here are listed in the Additional file 2. *Pfu* DNA polymerase was purchased from Solgent (Daejeon, Korea). Restriction enzymes and T4 DNA ligase were obtained from New England Biolabs (Ipswich, MA) and Roche (Mannheim, Germany), respectively. The genomic DNA of *E. coli* W3110 was amplified to overexpress the target genes using the *Pfu* polymerase and PCR primers (Additional file 2). The PCR product was then digested with *SacI* and *XbaI*, and ligated into p15SpeC at the same restriction sites downstream of the *tac* promoter *.*

### Fermentation

Batch cultivation was conducted at 37°C in a 6.6 L jar fermentor (Bioflo 3000; New Brunswick Scientific Co., Edison, NJ) containing 2 L R/2 medium supplemented with 10 g/L glucose and 3 g/L (NH_4_)_2_SO_4_. The R/2 medium (pH 6.8) contained (per liter): 2 g (NH_4_)_2_HPO_4_, 6.75 g KH_2_PO_4_, 0.85 g citric acid, and 0.7 g MgSO_4_·7H_2_O. In addition, 5 mL/L of a trace metal stock solution [35] was added. The trace metal solution contained per liter of 5 M HCl: 10 g FeSO_4_·7H_2_O, 2.25 g ZnSO_4_·7H_2_O, 1 g CuSO_4_·5H_2_O, 0.5 g MnSO_4_·5H_2_O, 0.23 g Na_2_B_4_O_7_·10H_2_O, 2 g CaCl_2_·2H_2_O, and 0.1 g (NH_4_)_6_Mo_7_O_24_. One milliliter of the overnight culture was transferred into a 300 mL Erlenmeyer flask containing 50 mL of the R/2 medium at 37°C and spun at 220 rpm in a shaking incubator (JEIOTech. Co. SI-900R). After obtaining an initial OD_600_ of 0.3, the seed cultures (200 mL) were introduced into the bioreactor for batch cultivation. The culture pH was maintained at 6.8 by the addition of 6 M KOH. The dissolved oxygen concentration was maintained at 20% air saturation by automatically adjusting the agitation speed. Under the comparable batch culture conditions, the single gene-overexpressing strains based on the *E. coli* XQ52 strain harboring p15SpeC, denoted as XQ52 (p15SpeC), with each target gene were tested by flask cultivation in duplicate using R/2 medium supplemented with 10 g/L glucose at 37 °C.

### Analytical procedures

Cell growth was estimated by measuring the optical density at 600 nm (OD_600_) using an Ultrospec 3000 spectrophotometer (Amersham Biosciences, Uppsala, Sweden). Glucose concentrations were measured using a glucose analyzer (model 2700 STAT; Yellow Springs Instrument, Yellow Springs, OH, USA). The concentrations of glucose and organic acids were determined by high-performance liquid chromatography (ProStar 210; Varian, Palo Alto, CA) equipped with UV/visible light (ProStar 320; Varian, Palo Alto, CA) and refractive index (Shodex RI-71, Tokyo, Japan) detectors. A MetaCarb 87H column (300 by 7.8 mm; Varian) was eluted isocratically with 0.01 NH_2_SO_4_ at 60°C at a flow rate of 0.4 mL/min.

The putrescine concentration was determined by derivatizing putrescine with *o-*phthaldialdehyde (OPA; Sigma, St. Louis, MO), and the *o-*phthaldialdehyde derivative was detected by high-performance liquid chromatography (1100 Series HPLC, Agilent Technologies, Palo Alto, CA) with UV detection, as described previously [34]. The OPA derivatization reagent was prepared as described previously [34,36,37]. Following the addition of the OPA reagent, the mixture was filtered through a 0.2 mm PVDF syringe filter (Whatman, Maidstone, UK), and the filtrate was immediately injected into the HPLC. A SUPELCO C18 column (cat# 504955; 5μm, 150 mm x 4.6 mm) was operated at 25°C with a 0.8 mL/min mobile phase flow rate. The mobile phase consisted of solution A (55% methanol in 0.1 M sodium acetate, pH 7.2) and solution B (methanol). The following gradient was applied (values given in vol%): 1–6 min, 100% A; 6–10 min, linear gradient of B from 0% to 30%; 10–15 min, linear gradient of B from 30% to 50%; 15–19 min, linear gradient of B from 50% to 100%; 19–23 min, 100% B; 23–25 min, linear gradient of B from 100% to 30%; 25–28 min, linear gradient of B from 30% to 0% [34]. The derivatized putrescine was detected at a wavelength of 230 nm using a variable wavelength detector (G1314A, Agilent Technologies).

## Results and discussion

### FVSEOF with GR constraints

Functionally related reactions can be grouped by genomic context and flux-converging pattern analyses [28]. Several reactions appeared to be related with one another based on genomic context analysis of conserved neighborhoods, gene fusions, and co-occurrence [28,29]. Flux-converging pattern analysis narrows the range of plausible flux values for metabolic reactions by examining the number of carbon atoms in metabolites that participate in reactions and the converging patterns of fluxes from a carbon source [28]. By controlling the simultaneous on/off activity (*C*_*on/off*_) and flux scale (*C*_*scale*_) of the metabolic reactions, based on FBA with GR constraints, the flux distributions in gene knockout mutants were accurately predicted [28]. In this study, the GR constraints were further applied to reactions related to the biosynthesis of a target chemical to improve the model accuracy (Figure 1 and the Additional file 3).

FVSEOF with GR constraints was implemented as follows (Figure 2). First, the theoretical minimal and maximal flux values for the target product formation were calculated using constraints-based flux analysis by minimizing and maximizing the target product formation rate with GR constraints. Second, again with GR constraints, the cell growth rate was maximized while gradually increasing the constraint value for the target product formation rate (our objective function which is artificially enforced) from a minimal to a theoretical maximum, as calculated from the first step. Finally, FVA was conducted by maximizing and minimizing the fluxes of all intracellular reactions under additional constraints, including GR constraints, the enforced production rate of the target chemical varied from a minimal to a maximal value, and a 95% optimal growth rate constraint for each step. The attainable flux ranges for the intracellular reactions were calculated under the imposed constraints for each of the three steps.

### Criteria for selecting gene amplification targets

Initial simulation results for FVSEOF with GR constraints were filtered based on rational criteria in an effort to select only the most effective amplification targets. The most important criterion was to identify gene amplification targets, the fluxes of which increased with the flux directed toward the target chemical. This procedure was implemented with quantitative values of *q*_*slope*_*V*_*avg*_, and *l*_*sol*_ (Figures 2 and 3). The flux bias (*V*_*avg*_) and flux capacity (*l*_*sol*_) indicate an average value for the maximal and minimal flux values and the length of the attainable flux ranges for a reaction, respectively [28]. Finally, the gene amplification candidates were evaluated by calculating the slope (*q*_*slope*_) of the *V*_*avg*_ flux for each metabolic reaction using linear regression analysis (Figure 2). Changes in the patterns of the reaction fluxes in response to incrementally increasing fluxes toward a target product were categorized into nine types based on combinations of positive and negative changes in *V*_*avg*_ and *l*_*sol*_ in order to facilitate the identification of amplification targets (Figure 3). Types 1, 2, and 3 displayed positive correlations with the amplification candidates for the production of a target chemical; types 4, 5, and 6 displayed negative correlations with the amplification candidates. Finally, types 7, 8, and 9 displayed no clear correlations with the production of a target chemical, based on *V*_*avg*_ (Figure 3). The reaction sets that were positively correlated (type 1, 2, and 3) were initially selected as amplification candidates.

Reaction sets belonging to type 1, 2 and 3, showing positive correlations with the enforced fluxes toward a target chemical, can then be further divided into strongly and weakly positive reactions (Figure 3 and Additional file 4). This step also allows narrowing down the candidates of gene amplification targets. The strongly positive reactions display a continuously increasing *V*_*avg*_ and a positive *q*_*slope*_ in response to the enhanced production of a target chemical, whereas weakly positive reactions show the same pattern, except for the presence of a partially negative *q*_*slope*_ (Additional file 4). Certainly, strongly positive reactions deserve primary attention as potential gene amplification targets.

The potential gene amplification candidates were prioritized by considering the *l*_*sol*_ value, which indicates the length between the maximal and minimal flux values of a metabolic reaction. Among the reactions that were positively correlated with the desired product, reactions with smaller values of *l*_*sol*_ received higher priorities because these reactions were more likely to display the predicted flux values than reactions with larger values of *l*_*sol*_. A final list of gene amplification targets obtained from the above procedure was then selected based on biological knowledge.

### Implementation of FVSEOF with GR constraints for shikimic acid production in *E. coli*

FVSEOF with GR constraints was employed to identify gene amplification targets for the enhanced production of an important aromatic chemical shikimic acid in *E. coli* (Figure 2). Shikimic acid is a key metabolic intermediate in the aromatic amino acid biosynthetic pathway. Shikimic acid and its derivatives are industrially important starting compounds for the production of several chemicals, such as phenols, herbicides, antibacterial agents, and the neuramidase inhibitor Tamiflu used for the treatment of influenza infections [38,39]. FVSEOF with GR constraints predicted that 11 reaction fluxes in the glycolysis (*glk* and *pps*), pentose phosphate pathway ( *rpi, talAB*, and *tktAB*), and the shikimic acid biosynthetic pathway ( *aroBaroDaroEaroFaroG*, and *aroH*) were potential amplification targets. The amplification of *aroBaroDaroEaroFaroGaroHtalABtktAglk*, and *pps* genes [38-46], which are the amplification targets predicted by FVSEOF with GR constraints, was previously reported to enhance the production of shikimic acid. The previous FSEOF method without FVA and GR constraints could not identify *pps* gene as an amplification target. FSEOF results without FVA and GR constraints did not show notable fluxes among metabolic reactions controlled by the *pps* gene in response to the enforced shikimic acid production rate; however, the FVSEOF method with GR constraints correctly predicted the *pps* gene as one of the amplification targets beneficial for the accumulation of phosphoenolpyruvate, an important precursor for the production of shikimic acid from pyruvate. This consistency partly demonstrated the power of utilizing FVA and GR constraints for predicting reliable amplification targets by FSEOF. In practice, the overexpression of phosphoenolpyruvate synthase encoded by the *pps* gene also increased the yield of precursors for the production of shikimic acid [43,45]. Thus, this strategy enabled the successful identification of gene amplification targets for the enhanced production of a primary metabolite, shikimic acid, in *E. coli*, in accordance with previous literature reports.

### Implementation of FVSEOF with GR constraints for enhanced putrescine production in *E. coli*

The general applicability of FVSEOF with GR constraints was examined by applying the method to putrescine production in *E. coli*. Putrescine (1,4-diaminobutane) is an important industrial precursor for the synthesis of polymers, pharmaceuticals, surfactants, and certain additives [34]. We confirmed the validity of the newly predicted gene amplification targets by comparison with the genes engineered in the previously reported putrescine-producing *E. coli* XQ52 (p15SpeC) strain [34]. FVSEOF with GR constraints predicted potential gene amplification targets among the reactions involved in glycolysis (*enopgm, gapA, fbaAB, tpiA, pgk, pykAF,* and *glk*), TCA cycle ( *icdacnAacnB,* and *gltA*), putrescine biosynthesis ( *gdhAargAargB, argC, argD, argE, speC,* and *speF*), and other pathways ( *ackA* and *ppc*). The predicted amplification targets ( *argB, argC, argD, argE, speC,* and *speF*) involved in the putrescine biosynthetic pathway were consistent with the mutations introduced in the *E. coli* XQ52 (p15SpeC) strain, as described in the previous report [34].

**Figure 4:**
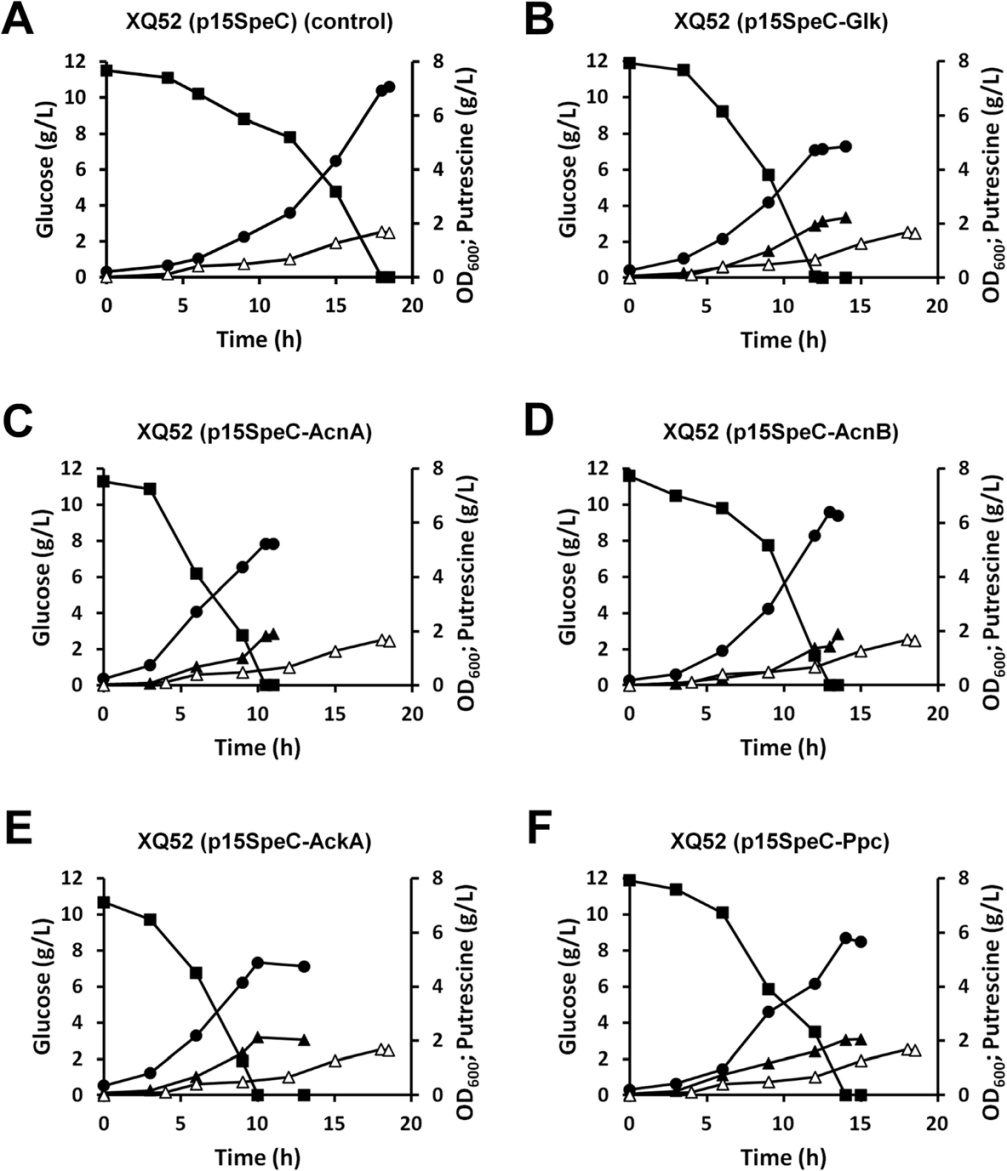
**Batch cultivation of the control and engineered*E. coli*strains producing putrescine.** ( **A**) XQ52 (p15SpeC) (control), ( **B**) XQ52 (p15SpeC-Glk), ( **C**) XQ52 (p15SpeC-AcnA), ( **D**) XQ52 (p15SpeC-AcnB), ( **E**) XQ52 (p15SpeC-AckA), and ( **F**) XQ52 (p15SpeC-Ppc) strains were tested for putrescine production. The symbols indicate: cell concentration measured by OD_600_ (**●**), glucose concentration (■), and putrescine concentration ( **▴**) for the engineered strains in ( **A**) to ( **F**), and putrescine concentration ( **▵**) for the XQ52 (p15SpeC) (control) strain in ( **A**).

The genes predicted to be relevant to the putrescine biosynthetic pathway were expected based on the pathway knowledge, and were intuitively obvious; hence, we focused on the effects associated with amplifying the predicted gene targets involved in other metabolic pathways in order to more rigorously validate FVSEOF with GR constraints. Accordingly, each of the predicted amplification targets was examined one by one by amplifying the gene dosage in the *E. coli* XQ52 (p15SpeC) strain (see Methods). Among these genes, the *glk, acnA, acnB, ackA,* and *ppc* gene, five out of the sixteen target genes, were found to attain increased putrescine yield when they were individually amplified in the *E. coli* XQ52 (p15SpeC) strain (Additional file 5). These strains, initially examined using flask cultivation, were further validated by batch cultivation at 37°C under aerobic condition (Figure 4 and see the Additional files 1 and 2). The recombinant *E. coli* XQ52 (p15SpeC) strain additionally expressing the *glk, acnA, acnB, ackA,* and *ppc* genes resulted in the production of 2.23, 1.90, 1.89, 2.04, and 2.06 g/L of putrescine, respectively, which are 20.5 % more than that (1.68 g/L) produced by the control strain on average (Figure 4). The yields of putrescine obtained with these strains were 0.223, 0.190, 0.189, 0.204, and 0.206 g putrescine per g glucose, which are again higher than that (0.168 g putrescine per g glucose) obtained with the control strain. Thus, FVSEOF with GR constraints could be successfully used to identify non-obvious gene amplification targets that enhance the production of putrescine in *E. coli*.

Other gene amplification targets identified by FVSEOF with GR constraints, which did not affect putrescine production in flask cultivation, also deserve discussion. These false-positive hits are most likely involved in biological processes that were not accurately captured in the genome-scale metabolic model. These ineffective genes, including *enopgmgapAfbaABtpiApgk*, and *pykAF* genes in glycolysis, and *icd* and *gltA* genes in TCA cycle might have been associated with transcriptional and translational regulations because the direct correlation between gene expressions and the metabolic fluxes was not observed. The fact that some of obvious gene amplification targets, such as *icd* gene responsible for biosynthesis of α-ketoglutarate, seem to be resistant to gene manipulations indicates that other biological variables may affect the effects of the gene amplifications. Potential variables include the plasmid copy number, gene dosage, optimal gene expression, and the gene expression method, either plasmid-based overexpression or chromosomal integration [47]. Although we improved the accuracy of the predicted gene targets by imposing GR constraints, these factors should be carefully considered in any implementation of the FVSEOF method with GR constraints [48].

## Conclusions

FVSEOF with GR constraints, which is an upgraded version of the FSEOF method, allows for the *in silico* identification of fluxes to be amplified for the enhanced production of target products. This method was conducted through the analysis of trends in reaction flux variability in response to varying the flux of target chemical production from initial to maximal flux values under GR constraints. The confidence with which amplification targets are identified may be increased by incorporating physiological data. This approach involves grouping functionally related reactions based on their genomic context and flux-converging pattern analyses. The interaction data may be obtained easily from public databases, and subjected to GR constraints. FVA was also performed to overcome the problems associated with multiple solutions for an optimal objective value. FVSEOF with GR constraints was shown to suggest successful metabolic engineering strategies (in particular, gene amplification) for the production of shikimic acid and putrescine in *E. coli*. In conclusion, the strategy reported here should be generally useful for developing industrial strains that display enhanced production of a target chemical.

## Abbreviations

FBA, Flux balance analysis; FSEOF, Flux scanning based on enforced objective flux; FVA, Flux variability analysis; FVSEOF, Flux variability scanning based on enforced objective flux; GR, Grouping reaction.

## Competing interests

The authors declare that they have no competing interests.

## Authors’ contributions

JMP, TYK, and SYL generated the ideas. JMP, HMP, WJK, TYK, and SYL designed the research. JMP, HMP, and WJK performed the research. JMP and HMP performed the analytical experiments. JMP, HMP, WJK, and HUK analyzed the data. JMP, HMP, WJK, HUK, and SYL wrote the paper. All authors read and approved the final manuscript.

## Supplementary Material

Additional file 1 **Bacterial strains and plasmids used in this study.** (PDF 142 kb) Click here for file

Additional file 2 **Oligonucleotides used in this study.** (PDF 101 kb) Click here for file

Additional file 3 **Genomic context and flux-converging pattern analyses for shikimic acid and putrescine production in*Escherichia coli.*** (PDF 143 kb) Click here for file

Additional file 4 **Analysis of flux patterns with partial variations.** (PDF 84 kb) Click here for file

Additional file 5 **Putrescine production yield (g putrescine/g glucose) for the single gene-overexpressing strains based on*E. coli*XQ52 (p15SpeC) strain by flask cultivation on R/2 medium supplemented with 10 g/L glucose at 37 °C.** (PDF 147 kb) Click here for file
