# Supplementary material for: Flux variability scanning based on enforced objective flux for identifying gene amplification targets
Source: BMC Syst Biol. 2012 Aug 21;6:106. doi: 10.1186/1752-0509-6-106 (PMC3443430; doi:10.1186/1752-0509-6-106)
Supplement: Additional file 1 — Bacterial strains and plasmids used in this study. (PDF 142 kb) [file 1752-0509-6-106-S1.pdf]

Additional file 1. Bacterial strains and plasmids used in this study

Strain or plasmid	Relevant characteristic(s)	References of sources
Strains		
E. coli W3110	Coli Genetic Stock Center strain(GGSC) No. 4474	CGSC[a]
WL3110	W3110 *ΔlacI*	Lab stock
TOP10	F^- mcrA Δ(*mrr-hsd*RMS-*mcr*BC) *¢80lac*Z Δ M15 D*lacX*74 *rec*A1 *ara*D139 Δ (*ara-leu*)7697 *gal*U *gal*K *rps*L (Str^R) *end*A1 *nup*G	Invitrogen[b]
XQ52	WL3110 *ΔspeE ΔspeG ΔargI ΔpuuPA PargECBH::Ptrc PspeF-potE::Ptrc PargD::Ptrc PspeC::Ptrc ΔrpoS*	Qian *et al.*(2009)
XQ52 (p15SpeC)	XQ52 harboring p15SpeC	This study
XQ52 (p15SpeC-Eno)	XQ52 harboring p15SpeC-Eno	This study
XQ52 (p15SpeC-Pgm)	XQ52 harboring p15SpeC-Pgm	This study
XQ52 (p15SpeC-GapA)	XQ52 harboring p15SpeC-GapA	This study
XQ52 (p15SpeC-FbaA)	XQ52 harboring p15SpeC-FbaA	This study
XQ52 (p15SpeC-FbaB)	XQ52 harboring p15SpeC-FbaB	This study
XQ52 (p15SpeC-TpiA)	XQ52 harboring p15SpeC-TpiA	This study
XQ52 (p15SpeC-Pgk)	XQ52 harboring p15SpeC-Pgk	This study
XQ52 (p15SpeC-PykA)	XQ52 harboring p15SpeC-PykA	This study
XQ52 (p15SpeC-PykF)	XQ52 harboring p15SpeC-PykF	This study
XQ52 (p15SpeC-Glk)	XQ52 harboring p15SpeC-Glk	This study
XQ52 (p15SpeC-IcdA)	XQ52 harboring p15SpeC-IcdA	This study
XQ52 (p15SpeC-AcnA)	XQ52 harboring p15SpeC-AcnA	This study
XQ52 (p15SpeC-AcnB)	XQ52 harboring p15SpeC-AcnB	This study
XQ52 (p15SpeC-GltA)	XQ52 harboring p15SpeC-GltA	This study
XQ52 (p15SpeC-AckA)	XQ52 harboring p15SpeC-AckA	This study
XQ52 (p15SpeC-Ppc)	XQ52 harboring p15SpeC-Ppc	This study
Plasmids		
pTac15K	Km^R, *tac* promoter, p15A ori, 4.0-kb	Qian *et al.*(2009)
p15SpeC	Km^R, *E. coli speC* gene in EcoRI-SacI site of pTac15K, 6.1-kb	This study
p15SpeC-Eno	Km^R, *E. coli eno* gene in SacI-XbaI site of p15SpeC, 7.1-kb	This study

p15SpeC-Pgm	Km^R, *E. coli pgm* gene in SacI-XbaI site of p15SpeC, 7.1-kb	This study
p15SpeC-GapA	Km^R, *E. coli gapA* gene in SacI-XbaI site of p15SpeC, 7.1-kb	This study
p15SpeC-FbaA	Km^R, *E. coli fbaA* gene in SacI-XbaI site of p15SpeC, 7.1-kb	This study
p15SpeC-FbaB	Km^R, *E. coli fbaB* gene in SacI-XbaI site of p15SpeC, 7.1-kb	This study
p15SpeC-TpiA	Km^R, *E. coli tpiA* gene in SacI-XbaI site of p15SpeC, 7.1-kb	This study
p15SpeC-Pgk	Km^R, *E. coli pgk* gene in SacI-XbaI site of p15SpeC, 7.1-kb	This study
p15SpeC-PykA	Km^R, *E. coli pykA* gene in SacI-XbaI site of p15SpeC, 7.1-kb	This study
p15SpeC-PykF	Km^R, *E. coli pykF* gene in SacI-XbaI site of p15SpeC, 7.1-kb	This study
p15SpeC-Glk	Km^R, *E. coli glk* gene in SacI-XbaI site of p15SpeC, 7.1-kb	This study
p15SpeC-IcdA	Km^R, *E. coli icdA* gene in SacI-XbaI site of p15SpeC, 7.1-kb	This study
p15SpeC-AcnA	Km^R, *E. coli acnA* gene in SacI-XbaI site of p15SpeC, 8.8-kb	This study
p15SpeC-AcnB	Km^R, *E. coli acnB* gene in SacI-XbaI site of p15SpeC, 8.7-kb	This study
p15SpeC-GltA	Km^R, *E. coli gltA* gene in SacI-XbaI site of p15SpeC, 7.1-kb	This study
p15SpeC-AckA	Km^R, *E. coli ackA* gene in SacI-XbaI site of p15SpeC, 7.3-kb	This study
p15SpeC-Ppc	Km^R, *E. coli ppc* gene in SacI-XbaI site of p15SpeC, 8.7-kb	This study

[a]Coli Genetic Stock Center, New Haven, CT.

[b]Invitrogen, Corp., Carlsbad, CA

Ap, ampicillin; Km, kanamycin; Str, streptomycin; R, resistance
