# Supplementary material for: Flux variability scanning based on enforced objective flux for identifying gene amplification targets
Source: BMC Syst Biol. 2012 Aug 21;6:106. doi: 10.1186/1752-0509-6-106 (PMC3443430; doi:10.1186/1752-0509-6-106)
Supplement: Additional file 2 — Oligonucleotides used in this study. (PDF 101 kb) [file 1752-0509-6-106-S2.pdf]

Additional file 2. Oligonucleotides used in this study

Name	Sequence(5' →3')*	Comments
speC_F	GATTGCGAATTCATGAAATCAATGAATATTGCCG	EcoRI
speC_R	GCATACGAGCTCTTACTTCAACACATAACCGTACAACC	SacI
acnA_F	ATAATGGAGCTCACAGGAAACAATGTCGTCAACCCTACGAGAAG	SacI
acnA_R	GGCAGGTCTAGATTACTTCAACATATTACGAATGACATAAT	XbaI
acnB_F	ATAATGGAGCTCACAGGAAACAGTGCTAGAAGAATACCGTAAGCAC	SacI
acnB_R	GCTATGTCTAGATTAAACCGCAGTCTGGAAAATCA	XbaI
ackA_F	GCTTTAGAGCTCACAGGAAACAATGTCGAGTAAGTTAGTACTGGTTCTG	SacI
ackA_R	ATTATGTCTAGATCAGGCAGTCAGGCGGCTCG	XbaI
glk_F	ATCATAGAGCTCACAGGAAACAATGACAAAGTATGCATTAGTCGG	SacI
glk_R	CGTCAGTCTAGATTACAGAATGTGACCTAAGGTCTGG	XbaI
ppc_F	ATCATAGAGCTCACAGGAAACAATGAACGAACAATATTCCGCA	SacI
ppc_R	GCTATGTCTAGATTAGCCGGTATTACGCATACCTG	XbaI
eno_F	GCTTAGAGCTCACAGGAAACAATGTCCAAAATCGTAAAAATCATC	SacI
eno_R	GCCGTCTCTAGATTATGCCTGGCCTTTGATCTC	XbaI
pgm_F	GTTATAGAGCTCACAGGAAACAATGGCAATCCACAATCGTGC	SacI
pgm_R	GCTGTGTCTAGATTACGCGTTTTTCAGAACTTCGCTAA	XbaI
gapA_F	ATCATAGAGCTCACAGGAAACAATGACTATCAAAGTAGGTATCAACGG	SacI
gapA_R	GCTGCGTCTAGATTATTTGGAGATGTGAGCGATCA	XbaI
fbaA_F	ATCATAGAGCTCACAGGAAACAATGTCTAAGATTTTTGATTTCGTAA	SacI
fbaA_R	GGTGGCTCTAGATTACAGAACGTCGATCGCGT	XbaI
fbaB_F	ATCATAGAGCTCACAGGAAACAATGACAGATATTGCGCAGTTG	SacI
fbaB_R	GAGCGTGTCTAGATCAGGCGATAGTAATTTTGCTATC	XbaI
tpiA_F	ATCATAGAGCTCACAGGAAACAATGCGACATCCTTTAGTGATGG	SacI
tpiA_R	GCTCGACTCTAGATTAAGCCTGTTTAGCCGCTTC	XbaI
pgk_F	ATCATAGAGCTCACAGGAAACAATGTCTGTAATTAAGATGACCGATC	SacI
pgk_R	GACTATGTCTAGATTACTTCTTAGCGCGCTCTTCG	XbaI
pykA_F	ATCATAGAGCTCACAGGAAACAATGTCCAGAAGGCTTCGCAG	SacI
pykA_R	GCTAGGGTCTAGATTACTCTACCGTTAAAATACGCGT	XbaI
pykF_F	ATCATAGAGCTCACAGGAAACAATGAAAAAGACCAAAATTGTTTG	SacI

pykF_R	GCTATGTCTAGATTACAGGACGTGAACAGATGCG	XbaI
icd_F	ACTTACGAGCTCACAGGAAACAATGGAAAGTAAAGTAGTTGTTCCG	SacI
icd_R	GCCGACTCTAGATTACATGTTTTCGATGATCGCG	XbaI
gltA_F	ATCATAGAGCTCACAGGAAACAATGGCTGATACAAAAGCAAAACT	SacI
gltA_R	GCTCCGTCTAGATTAACGCTTGATATCGCTTTTAAAG	XbaI

*Restriction sites are underlined.
