# Supplementary material for: Flux variability scanning based on enforced objective flux for identifying gene amplification targets
Source: BMC Syst Biol. 2012 Aug 21;6:106. doi: 10.1186/1752-0509-6-106 (PMC3443430; doi:10.1186/1752-0509-6-106)
Supplement: Additional file 3 — Genomic context and flux-converging pattern analyses for shikimic acid and putrescine production inEscherichia coli. (PDF 143 kb) [file 1752-0509-6-106-S3.pdf]

Additional file 3. Genomic context and flux-converging pattern analyses for shikimic acid and putrescine production in *Escherichia coli*

Reaction	Gene	C_xJ_y (Glucose)	Enzyme	Metabolism	EC number	Score					
						>0.95	>0.9	>0.85	>0.8	>0.75	>0.7
**Central metabolism**											
The detailed information of grouped reactions in central metabolism of *E. coli* is represented in Supporting Information of Park et al. (2010)[1].											
**For shikimic acid production**											
DDPA	*aroG*<br>*aroH*<br>*aroF*	C_7J_B	3-deoxy-D-arabino-heptulosonate 7-phosphate synthetase	Shikimate biosynthetic pathway	2.5.1.54			*tyrA*			
DHQS	*aroB*	C_7J_B	3-dehydroquinate synthase	Shikimate biosynthetic pathway	4.2.3.4	*aroK, aroL, aroA, aroE, aroD*	*aroC, ydiB*			*kdsA, alaS*	*ileS, hofQ, pheA, mrcA, pfs, ubiE*
DHQD	*aroD*	C_7J_B	3-dehydroquinate dehydratase	Shikimate biosynthetic pathway	4.2.1.10	*ydiB, aroB*	*aroA, aroE, ydiN, aroK*	*ydiM*		*aroC*	
SHK3Dr	*aroE*<br>*ydiB*	C_7J_B	shikimate dehydrogenase	Shikimate biosynthetic pathway	1.1.1.25	*aroB, aroA*	*aroC, aroD*	*yrdB, rimN, yrdD*		*rimL, aroK*	*smf, smg, aroL*
**For putrescine production**											

GLUDy	*gdhA*	C_5J_{CE}/ C_5J_{DE}/ C_5J_B	glutamate dehydrogenase	Putrescine biosynthetic pathway	1.4.1.4						
ACGS	*argA*	C_7J_{CE}/ C_7J_{DE}/ C_7J_B	N-acetylglutamate synthase	Putrescine biosynthetic pathway	2.3.1.1	*argC*	*argH*	*argB*	*argE*		*topA*
ACGK	*argB*	C_7J_{CE}/ C_7J_{DE}/ C_7J_B	acetylglutamate kinase	Putrescine biosynthetic pathway	2.7.2.8	*argC*	*argH, astC, argD*	*argA*	*argE, argI*	*argF, argG*	*carA*
AGPR	*argC*	C_7J_{CE}/ C_7J_{DE}/ C_7J_B	N-acetyl-g-glutamyl-phosphate reductase	Putrescine biosynthetic pathway	1.2.1.38	*argB, argA*	*argH, astC, argD*			*rplM, rpsI, argG, argI, argF, argR*	*argE, rimK*
ACOTA	*argD*	C_7J_{CE}/ C_7J_{DE}/ C_7J_B	acetylornithine transaminase	Putrescine biosynthetic pathway	2.6.1.11		*argC, argB*				
ACODA	*argE*	C_7J_{CE}/ C_7J_{DE}/ C_7J_B	acetylornithine deacetylase	Putrescine biosynthetic pathway	3.5.1.16				*argB, argC, argA*		
ORNDC	*speC*<br>*speF*	C_5J_{CE}/ C_5J_{DE}/ C_5J_B	Ornithine decarboxylase	Putrescine biosynthetic pathway	4.1.1.17				*potE*<br>*potE*		

References

1. Park JM, Kim TY, Lee SY: **Prediction of metabolic fluxes by incorporating genomic context and flux-converging pattern analyses.** *Proc Natl Acad Sci U S A* 2010, **107:**14931-14936.
