# Supplementary material for: Flux variability scanning based on enforced objective flux for identifying gene amplification targets
Source: BMC Syst Biol. 2012 Aug 21;6:106. doi: 10.1186/1752-0509-6-106 (PMC3443430; doi:10.1186/1752-0509-6-106)
Supplement: Additional file 4 — Analysis of flux patterns with partial variations. (PDF 84 kb) [file 1752-0509-6-106-S4.pdf]

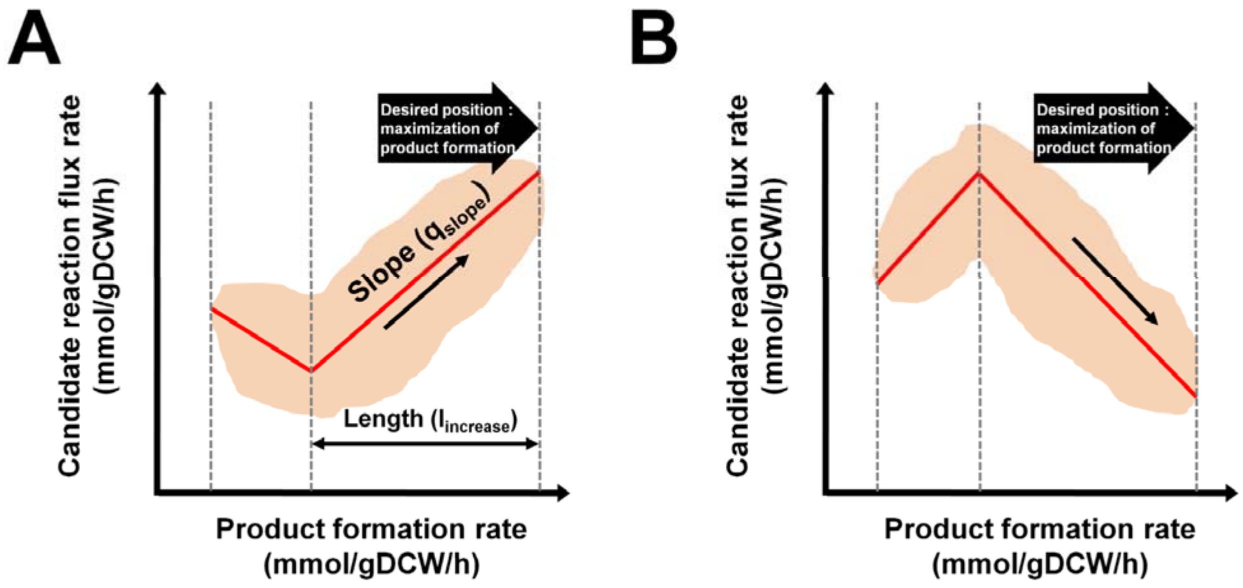

Additional file 4. Analysis of flux patterns with partial variations. (A) A case that has overall a positive correlation between the candidate reaction flux rate and the product formation rate. In this case, there exists a section indicating a desired position of maximizing product formation rate. This case is therefore considered as one of gene amplification candidates. This case is deemed as "weakly positive reactions" as it has the section of partially negative q_{slope}, despite the general profile of V_{avg} increasing. The maximization of candidate reaction flux in this case (amplification of corresponding gene) can have a beneficial effect on maximizing the product formation rate. Reactions corresponding to this case can be prioritized by considering the slope value of the increasing flux pattern (q_{slope}) and the length of the section indicating the desired position ($l_{increase}$). The large values of q_{slope} and $l_{increase}$ were considered to be beneficial for gene amplification targets. (B) A case that has a section of partially positive q_{slope} but has overall a negative correlation between the candidate reaction flux rate and the product formation rate (decreased change of flux pattern). This case was discarded from the candidate list.
