# Supplementary material for: Flux variability scanning based on enforced objective flux for identifying gene amplification targets
Source: BMC Syst Biol. 2012 Aug 21;6:106. doi: 10.1186/1752-0509-6-106 (PMC3443430; doi:10.1186/1752-0509-6-106)
Supplement: Additional file 5 — Putrescine production yield (g putrescine/g glucose) for the single gene-overexpressing strains based onE. coliXQ52 (p15SpeC) strain by flask cultivation on R/2 medium supplemented with 10 g/L glucose at 37 °C. (PDF 147 kb) [file 1752-0509-6-106-S5.pdf]

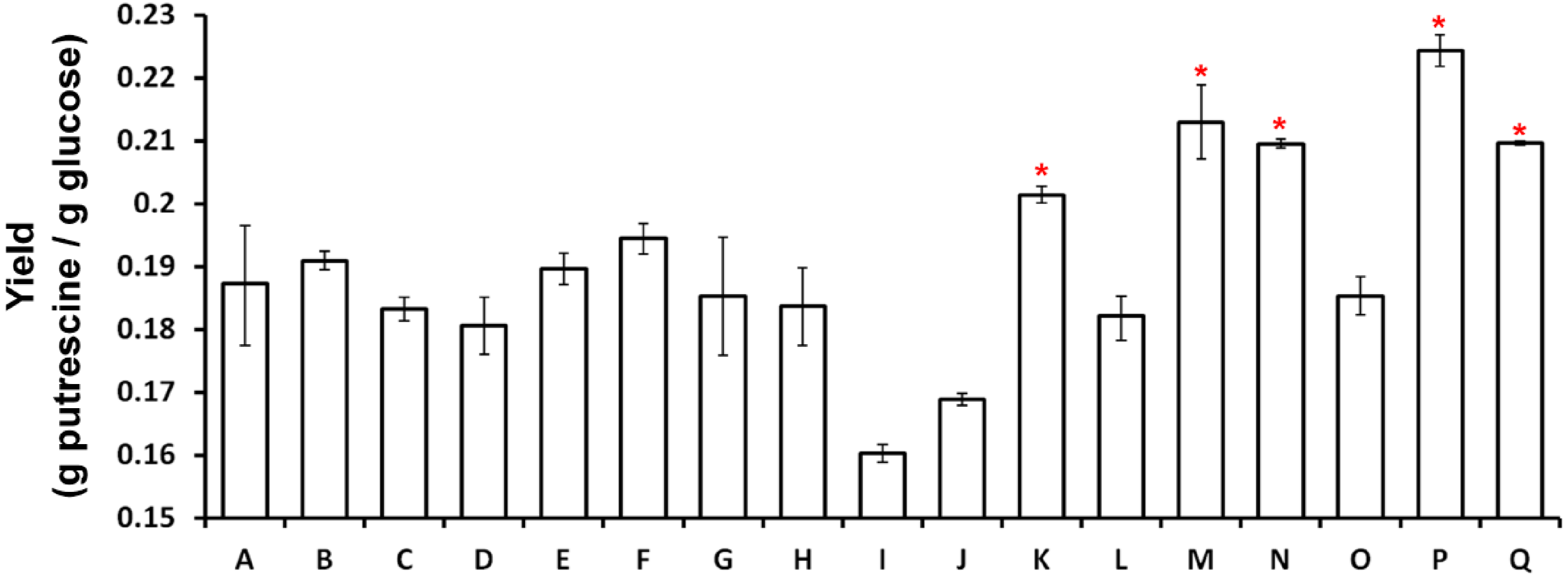

Additional file 5. Putrescine production yield (g putrescine/g glucose) for the single gene-overexpressing strains based on *E. coli* XQ52 (p15SpeC) strain by flask cultivation on R/2 medium supplemented with 10 g/L glucose at 37℃. (A) XQ52 (p15SpeC) (control), (B) XQ52 (p15SpeC-Eno), (C) XQ52 (p15SpeC-Pgm), (D) XQ52 (p15SpeC-GapA), (E) XQ52 (p15SpeC-FbaA), (F) XQ52 (p15SpeC-FbaB), (G) XQ52 (p15SpeC-TpiA), (H) XQ52 (p15SpeC-Pgk), (I) XQ52 (p15SpeC-PykA), (J) XQ52 (p15SpeC-PykF), (K) XQ52 (p15SpeC-Glk), (L) XQ52 (p15SpeC-Icd), (M) XQ52 (p15SpeC-AcnA), (N) XQ52 (p15SpeC-AcnB), (O) XQ52 (p15SpeC-GltA), (P) XQ52 (p15SpeC-AckA), and (Q) XQ52 (p15SpeC-Ppc) strains. The '*' indicates the selected strains for further validation by batch cultivation, which show improved results in comparison with the control strain by considering error bar. All the experiments were performed in duplicate.
